# The top 100 most-cited articles in perioperative stroke: a bibliometric analysis

**DOI:** 10.1097/JS9.0000000000001946

**Published:** 2024-07-11

**Authors:** Xiaoting Shi, Li Dai, Shaosheng Wu

**Affiliations:** Department of Anesthesiology, Changde Hospital, Xiangya School of Medical, Central South University (The first people’s hospital of Changde City), Changde, People’s Republic of China

Highlights

- This study analyzed the top 100 cited articles in the field of perioperative stroke using bibliometric methods.
- Main research themes in perioperative stroke were identified, along with notable institutions and countries.
- This analysis provided valuable insights into the prevailing trends and gaps in research, guiding the direction of future investigations and interventions in this important clinical area.

*Dear Editor,*

Perioperative stroke, a serious complication in surgical patients, has captured global attention. Defined as cerebral infarction within 30 days postsurgery^1^, its incidence among adults undergoing noncardiac procedures ranges from 0.1 to 0.7%^2^. Additionally, covert strokes affect ~7% of similar patients aged 65 and older^3^. According to a recent study by Wu *et al*.^4^ in the International Journal of Surgery, a history of stroke significantly raises the risk of mortality in patients undergoing noncardiac surgery. Delayed detection of perioperative stroke impedes effective intervention, resulting in 77% of these patients suffering mortality or severe disability upon long-term care discharge^5^. This highlights the urgent need for deeper insights into perioperative stroke despite extensive existing research.

We searched the Web of Science core database on 28 April 2024. Data extraction was conducted using the following search strategy: Topic Subject (TS)= (perioperative stroke OR postoperative stroke OR intraoperative stroke), besides the language is English. A total of 14 506 manuscripts were retrieved, and articles were sorted in descending order of total citations. Consequently, the top 100 most cited articles on perioperative stroke were identified and analyzed (81 original articles and 19 reviews or guidelines). Bibliometric indicators, including publication year, annual total citation count, annual average citation count (ACY), publication journal, authors, country/region, institution, research type, and keywords, were extracted. The retrieved data were analyzed for trends and hotspots using R4.3.3 software (Biblioshiny), the bibliometric website (http://bibliometric.com/), and CiteSpace 6.3.R1.

The top 100 cited articles were published between 1991 and 2018, the top 10 countries that significantly contributed to the field of in Perioperative Stroke are shown in Figure 1 A. The highest number of articles was published in 2015 (*n*=9), as shown in Supplementary Table 1 (Supplemental Digital Content 1, http://links.lww.com/JS9/D53). The total citation count for these articles ranged from 320 to 3943, with a median total of 462 citations. Among them, the most cited paper, authored by Professor Walker Md in 1995, explored the efficacy of adding carotid endarterectomy to aggressive therapy in reducing the incidence of stroke in asymptomatic carotid stenosis patients. The most recent article in this top 100 was published in 2018, presenting a retrospective study on a deep learning algorithm for detecting key findings in head CT scans. A total of 27 countries contributed to these top 100 articles, with five countries contributing four or more articles each. Notably, the United States led with 49 top 100 articles and 35 435 citations, underscoring its significant scientific contribution. The corporation map of countries shows Canada and the USA had tight corporations, followed by UK as shown in Figure 1B. A total of 525 scientists from around the world contributed to the Top 100 articles, with seven first authors having published a maximum of two articles. In total, over 331 different institutions participated, with McMaster University from Canada leading with 12 top 100 articles and 7594 citations. These articles were published across 36 different journals, with The New England Journal of Medicine having the most publications (*n*=15), garnering 11 918 citations. The majority of the manuscripts were original articles (*n*=81), among which 52 (64.2%) reported interventional clinical studies (i.e. clinical trials), 28 (34.6%) reported observational clinical studies, and 1 (1.2%) reported skilled walking in the ladder rung walking test with cortical and subcortical lesions in rats. Among these, four articles mention hemorrhagic stroke, with only one article specifically focusing on hemorrhagic stroke. The majority, comprising 96 articles, are concentrated on ischemic stroke.

**Figure 1:**
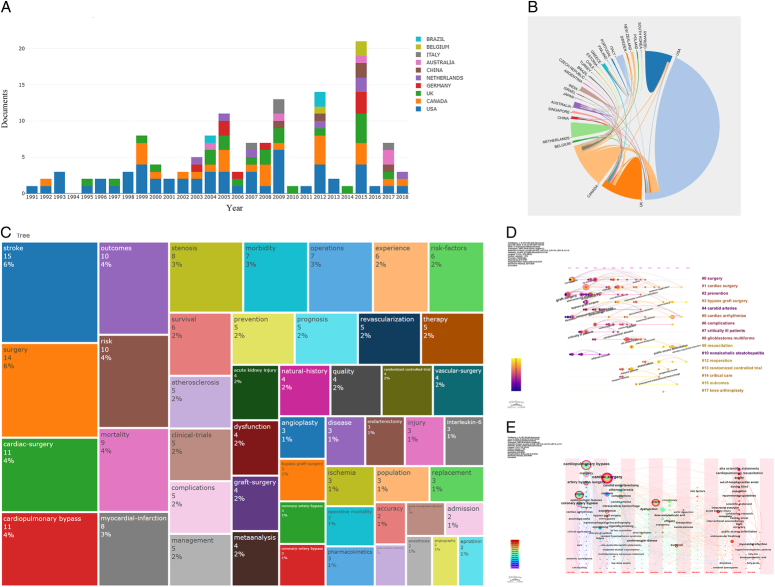
The research trends and hotspots map (A) Annual publication of the top 10 countries in the research of perioperative stroke (B) The corporation map among the countries (C) Tree map chart of the 50 most frequently used keyword terms in original articles from 1991 to 2018 (D) The timeline view of co-cited keywords of perioperative stroke. (E) The timezone view of co-cited keywords of perioperative stroke.

The five most frequently used keywords are: ‘stroke’, ‘surgery’, ‘cardiac-surgery’, ‘cardiopulmonary bypass’, and ‘outcome’ (Fig. 1C). Keyword co-occurrence analysis yielded 430 nodes and 1608 links, with clustering analysis categorizing these keywords into 16 clusters (Fig. 1D). Each cluster is depicted as a horizontal timeline from left to right. The timeline map of co-cited literature indicates that cardiac surgery (#1), bypass graft surgery (#3), cardiac arrhythmias (#5), resuscitation (#9), reoperation (#12), and outcome (#15) are presently the most prominent research topics. The analysis of keyword frequency across different time periods illustrates a significant shift in focus (Fig. 1E). Initially, the emphasis was predominantly on surgical techniques and immediate outcomes. Over time, this focus has broadened to prioritize the prevention of long-term complications and the enhancement of research methodologies. This transition highlights a growing interest in not only addressing immediate clinical needs but also in understanding long-term impacts and improving the overall quality of scientific inquiry in the field. Such insights are crucial for informing future research directions and for developing more comprehensive approaches to cardiovascular health.

The bibliometric analysis scrutinized the top 100 most cited articles in perioperative stroke, revealing the United States as the foremost contributor in terms of article count, scientists, and affiliated institutions. The New England Journal of Medicine emerged as the primary publisher of articles within this elite selection. Interventions clinical studies predominated as the primary research type in this domain. Ongoing research endeavors concentrate on pinpointing high-risk stroke populations, refining preoperative medication protocols, optimizing intraoperative management strategies, and enhancing postoperative stroke diagnosis and treatment modalities. These findings offer invaluable insights into the landscape of perioperative stroke research, facilitating a deeper understanding of research quality and trends while maximizing the utility of classical literature in this field.

## Ethical approval

This research letter to the editor did not require ethical approval or consent to participate.

## Consent

No applicable.

## Source of funding

None.

## Author contribution

S.X.T. and W.S.S.: conceptualization; S.X.T. and D.L.: data acquisition; S.X.T.: statistical analysis; D.L.: creation of figure; S.X.T. and W.S.S.: writing – review and editing and supervision.

## Conflicts of interest disclosure

The authors declare no competing interests.

## Research registration unique identifying number (UIN)

None.

## Guarantor

All authors.

## Data available statement

The data presented in this study are available on request from the corresponding author.

## Provenance and peer review

Not commissioned, externally peer-reviewed.

## Assistance with the study

None.

## Supplementary Material
