# Supplementary material for: The top 100 most-cited articles in perioperative stroke: a bibliometric analysis
Source: Int J Surg. 2024 Jul 11;111(1):1492–4. doi: 10.1097/JS9.0000000000001946 (PMC11745590; doi:10.1097/JS9.0000000000001946)
Supplement: Supplementary file 1 [file js9-111-1492-s001.doc]

**The Top 100 articles in the field of perioperative stroke research**

| **Rank** | **Author** | **Title** | **Journal** | **Year** | **Tc** | **AC/Y** |
| --- | --- | --- | --- | --- | --- | --- |
| 1 | Walker Md et al.. | Endarterectomy For Asymptomatic Carotid-Artery Stenosis | Jama-Journal Of The American Medical Association | 1995 | 3943 | 131.43 |
| 2 | Stummer W et al.. | Fluorescence-Guided Surgery With 5-Aminolevulinic Acid For Resection Of Malignant Glioma: A Randomised Controlled Multicentre Phase Iii Trial | Lancet Oncology | 2006 | 2388 | 125.68 |
| 3 | Yadav Js et al.. | Protected Carotid-Artery Stenting Versus Endarterectomy In High-Risk Patients | New England Journal Of Medicine | 2004 | 2038 | 97.05 |
| 4 | Halliday A et al.. | Prevention Of Disabling And Fatal Strokes By Successful Carotid Endarterectomy In Patients Without Recent Neurological Symptoms: Randomised Controlled Trial | Lancet | 2004 | 1804 | 85.90 |
| 5 | Devereaux Pj et al.. | Effects Of Extended-Release Metoprolol Succinate Inpatients Undergoing Non-Cardiac Surgery (Poise Trial):: A Randomised Controlled Trial | Lancet | 2008 | 1463 | 86.06 |
| 6 | Roach Gw et al.. | Adverse Cerebral Outcomes After Coronary Bypass Surgery | New England Journal Of Medicine | 1996 | 1407 | 48.52 |
| 7 | Miller Lw et al.. | Use Of A Continuous-Flow Device In Patients Awaiting Heart Transplantation | New England Journal Of Medicine | 2007 | 1303 | 72.39 |
| 8 | Blackshear Jl et al.. | Appendage Obliteration To Reduce Stroke In Cardiac Surgical Patients With Atrial Fibrillation | Annals Of Thoracic Surgery | 1996 | 1110 | 38.28 |
| 9 | Chung Mk et al.. | C-Reactive Protein Elevation In Patients With Atrial Arrhythmias - Inflammatory Mechanisms And Persistence Of Atrial Fibrillation | Circulation | 2001 | 1100 | 45.83 |
| 10 | Hobson Rw et al.. | Efficacy Of Carotid Endarterectomy For Asymptomatic Carotid Stenosis | New England Journal Of Medicine | 1993 | 1084 | 33.88 |
| 11 | Rothwell Pm et al.. | Endarterectomy For Symptomatic Carotid Stenosis In Relation To Clinical Subgroups And Timing Of Surgery | Lancet | 2004 | 1032 | 49.14 |
| 12 | Murphy Gj et al.. | Increased Mortality, Postoperative Morbidity, And Cost After Red Blood Cell Transfusion In Patients Having Cardiac Surgery | Circulation | 2007 | 994 | 55.22 |
| 13 | Oppenheimer Sm et al.. | Cardiovascular Effects Of Human Insular Cortex Stimulation | Neurology | 1992 | 986 | 29.88 |
| 14 | Pollack Cv et al.. | Idarucizumab For Dabigatran Reversal | New England Journal Of Medicine | 2015 | 959 | 95.90 |
| 15 | O'Brien Sm et al.. | The Society Of Thoracic Surgeons 2008 Cardiac Surgery Risk Models: Part 2-Isolated Valve Surgery | Annals Of Thoracic Surgery | 2009 | 958 | 59.88 |
| 16 | Chertow Gm et al.. | Independent Association Between Acute Renal Failure And Mortality Following Cardiac Surgery | American Journal Of Medicine | 1998 | 940 | 34.81 |
| 17 | Svensson Lg et al.. | Experience With 1509 Patients Undergoing Thoracoabdominal Aortic Operations | Journal Of Vascular Surgery | 1993 | 928 | 29.00 |
| 18 | Nolan Jp et al.. | Post-Cardiac Arrest Syndrome: Epidemiology, Pathophysiology, Treatment, And Prognostication A Scientific Statement From The International Liaison Committee On Resuscitation; The American Heart Association Emergency Cardiovascular Care Committee; The Council On Cardiovascular Surgery And Anesthesia; The Council On Cardiopulmonary, Perioperative, And Critical Care; The Council On Clinical Cardiology; The Council On Stroke | Resuscitation | 2008 | 873 | 51.35 |
| 19 | Ferguson Gg et al.. | The North American Symptomatic Carotid Endarterectomy Trial - Surgical Results In 1415 Patients | Stroke | 1999 | 837 | 32.19 |
| 20 | Engel J et al.. | Early Surgical Therapy For Drug-Resistant Temporal Lobe Epilepsy A Randomized Trial | Jama-Journal Of The American Medical Association | 2012 | 829 | 63.77 |
| 21 | Perkins Gd et al.. | Cardiac Arrest And Cardiopulmonary Resuscitation Outcome Reports: Update Of The Utstein Resuscitation Registry Templates For Out-Of-Hospital Cardiac Arrest A Statement For Healthcare Professionals From A Task Force Of The International Liaison Committee On Resuscitation | Circulation | 2015 | 801 | 80.10 |
| 22 | Stummer W et al.. | Extent Of Resection And Survival On Glioblastoma Multiforme-Identification Of And Adiustment For Bias | Neurosurgery | 2008 | 791 | 46.53 |
| 23 | Nussmeier Na et al.. | Complications Of The Cox-2 Inhibitors Parecoxib And Valdecoxib After Cardiac Surgery | New England Journal Of Medicine | 2005 | 770 | 38.50 |
| 24 | Krinsley Js et al.. | Effect Of An Intensive Glucose Management Protocol On The Mortality Of Critically Ill Adult Patients | Mayo Clinic Proceedings | 2004 | 767 | 36.52 |
| 25 | Douketis Jd et al.. | Perioperative Bridging Anticoagulation In Patients With Atrial Fibrillation | New England Journal Of Medicine | 2015 | 739 | 73.90 |
| 26 | Mayberg Mr et al.. | Carotid Endarterectomy And Prevention Of Cerebral-Ischemia In Symptomatic Carotid Stenosis | Jama-Journal Of The American Medical Association | 1991 | 725 | 21.32 |
| 27 | Creswell Ll et al.. | Hazards Of Postoperative Atrial Arrhythmias | Annals Of Thoracic Surgery | 1993 | 706 | 22.06 |
| 28 | Brown Jm et al.. | Isolated Aortic Valve Replacement In North America Comprising 108,687 Patients In 10 Years: Changes In Risks, Valve Types, And Outcomes In The Society Of Thoracic Surgeons National Database | Journal Of Thoracic And Cardiovascular Surgery | 2009 | 704 | 44.00 |
| 29 | Gokce N et al.. | Risk Stratification For Postoperative Cardiovascular Events Via Noninvasive Assessment Of Endothelial Function - A Prospective Study | Circulation | 2002 | 676 | 29.39 |
| 30 | Gan Tj et al.. | Goal-Directed Intraoperative Fluid Administration Reduces Length Of Hospital Stay After Major Surgery | Anesthesiology | 2002 | 670 | 29.13 |
| 31 | Willinsky Ra et al.. | Neurologic Complications Of Cerebral Angiography: Prospective Analysis Of 2,899 Procedures And Review Of The Literature | Radiology | 2003 | 641 | 29.14 |
| 32 | Halliday A et al.. | 10-Year Stroke Prevention After Successful Carotid Endarterectomy For Asymptomatic Stenosis (Acst-1): A Multicentre Randomised Trial | Lancet | 2010 | 638 | 42.53 |
| 33 | Douketis Jd et al.. | The Perioperative Management Of Antithrombotic Therapy | Chest | 2008 | 623 | 36.65 |
| 34 | Brinjikji W et al.. | Endovascular Treatment Of Intracranial Aneurysms With Flow Diverters A Meta-Analysis | Stroke | 2013 | 620 | 51.67 |
| 35 | Murkin Jm et al.. | Monitoring Brain Oxygen Saturation During Coronary Bypass Surgery: A Randomized, Prospective Study | Anesthesia And Analgesia | 2007 | 552 | 30.67 |
| 36 | Almassi Gh et al.. | Atrial Fibrillation After Cardiac Surgery - A Major Morbid Event? | Annals Of Surgery | 1997 | 551 | 19.68 |
| 37 | Murphy Gj et al.. | Liberal Or Restrictive Transfusion After Cardiac Surgery | New England Journal Of Medicine | 2015 | 545 | 54.50 |
| 38 | Metz Ga et al.. | Cortical And Subcortical Lesions Impair Skilled Walking In The Ladder Rung Walking Test: A New Task To Evaluate Fore- And Hindlimb Stepping, Placing, And Co-Ordination | Journal Of Neuroscience Methods | 2002 | 538 | 23.39 |
| 39 | Sinclair S et al.. | Intraoperative Intravascular Volume Optimisation And Length Of Hospital Stay After Repair Of Proximal Femoral Fracture: Randomised Controlled Trial | Bmj-British Medical Journal | 1997 | 522 | 18.64 |
| 40 | Connolly Sj et al.. | Effects Of Physiologic Pacing Versus Ventricular Pacing On The Risk Of Stroke And Death Due To Cardiovascular Causes | New England Journal Of Medicine | 2000 | 520 | 20.80 |
| 41 | Hausenloy Dj et al.. | Remote Ischemic Preconditioning And Outcomes Of Cardiac Surgery | New England Journal Of Medicine | 2015 | 518 | 51.80 |
| 42 | Lamy A et al.. | Off-Pump Or On-Pump Coronary-Artery Bypass Grafting At 30 Days | New England Journal Of Medicine | 2012 | 512 | 39.38 |
| 43 | Svensson Lg et al.. | Deep Hypothermia With Circulatory Arrest - Determinants Of Stroke And Early Mortality In 656 Patients | Journal Of Thoracic And Cardiovascular Surgery | 1993 | 502 | 15.69 |
| 44 | Villareal Rp et al.. | Postoperative Atrial Fibrillation And Mortality After Coronary Artery Bypass Surgery | Journal Of The American College Of Cardiology | 2004 | 493 | 23.48 |
| 45 | Liapis Cd et al.. | Esvs Guidelines. Invasive Treatment For Carotid Stenosis: Indications, Techniques | European Journal Of Vascular And Endovascular Surgery | 2009 | 482 | 30.13 |
| 46 | Diethrich Eb et al.. | Stenting In The Carotid Artery: Initial Experience In 110 Patients | Journal Of Endovascular Surgery | 1996 | 479 | 16.52 |
| 47 | Marceau P et al.. | Liver Pathology And The Metabolic Syndrome X In Severe Obesity | Journal Of Clinical Endocrinology \& Metabolism | 1999 | 475 | 18.27 |
| 48 | Chilamkurthy S et al.. | Deep Learning Algorithms For Detection Of Critical Findings In Head Ct Scans: A Retrospective Study | Lancet | 2018 | 471 | 67.29 |
| 49 | Ricotta Jj et al.. | Updated Society For Vascular Surgery Guidelines For Management Of Extracranial Carotid Disease | Journal Of Vascular Surgery | 2011 | 469 | 33.50 |
| 50 | Meybohm P et al.. | A Multicenter Trial Of Remote Ischemic Preconditioning For Heart Surgery | New England Journal Of Medicine | 2015 | 467 | 46.70 |
| 51 | Bucerius J et al.. | Stroke After Cardiac Surgery: A Risk Factor Analysis Of 16,184 Consecutive Adult Patients | Annals Of Thoracic Surgery | 2003 | 457 | 20.77 |
| 52 | Mythen Mg et al.. | Perioperative Plasma-Volume Expansion Reduces The Incidence Of Gut Mucosal Hypoperfusion During Cardiac-Surgery | Archives Of Surgery | 1995 | 457 | 15.23 |
| 53 | Gowda Rm et al.. | Cardiac Papillary Fibroelastoma: A Comprehensive Analysis Of 725 Cases | American Heart Journal | 2003 | 449 | 20.41 |
| 54 | Puskas Jd et al.. | Off-Pump Coronary Artery Bypass Grafting Provides Complete Revascularization With Reduced Myocardial Injury, Transfusion Requirements, And Length Of Stay: A Prospective Randomized Comparison Of Two Hundred Unselected Patients Undergoing Off-Pump Versus Conventional Coronary Artery Bypass Grafting | Journal Of Thoracic And Cardiovascular Surgery | 2003 | 449 | 20.41 |
| 55 | Alexander Kp et al.. | Outcomes Of Cardiac Surgery In Patients Age ≥80 Years:: Results From The National Cardiovascular Network | Journal Of The American College Of Cardiology | 2000 | 443 | 17.72 |
| 56 | Buth J et al.. | Neurologic Complications Associated With Endovascular Repair Of Thoracic Aortic Pathology: Incidence And Risk Factors. A Study From The European Collaborators On Stent/Graft Techniques For Aortic Aneurysm Repair (Eurostar) Registry | Journal Of Vascular Surgery | 2007 | 441 | 24.50 |
| 57 | Wakeling Hg et al.. | Intraoperative Oesophageal Doppler Guided Fluid Management Shortens Postoperative Hospital Stay After Major Bowel Surgery | British Journal Of Anaesthesia | 2005 | 440 | 22.00 |
| 58 | Chu Dk et al.. | Mortality And Morbidity In Acutely Ill Adults Treated With Liberal Versus Conservative Oxygen Therapy (Iota): A Systematic Review And Meta-Analysis | Lancet | 2018 | 438 | 62.57 |
| 59 | Zmistowski B et al.. | Periprosthetic Joint Infection Increases The Risk Of One-Year Mortality | Journal Of Bone And Joint Surgery-American Volume | 2013 | 438 | 36.50 |
| 60 | Healey Js et al.. | Periprocedural Bleeding And Thromboembolic Events With Dabigatran Compared With Warfarin Results From The Randomized Evaluation Of Long-Term Anticoagulation Therapy (Re-Ly) Randomized Trial | Circulation | 2012 | 429 | 33.00 |
| 61 | Cappato R et al.. | Prevalence And Causes Of Fatal Outcome In Catheter Ablation Of Atrial Fibrillation | Journal Of The American College Of Cardiology | 2009 | 421 | 26.31 |
| 62 | Siscovick Ds et al.. | Omega-3 Polyunsaturated Fatty Acid (Fish Oil) Supplementation And The Prevention Of Clinical Cardiovascular Disease: A Science Advisory From The American Heart Association | Circulation | 2017 | 417 | 52.13 |
| 63 | Durazzo Aes et al.. | Reduction In Cardiovascular Events After Vascular Surgery With Atorvastatin: A Randomized Trial | Journal Of Vascular Surgery | 2004 | 413 | 19.67 |
| 64 | Carnes Ca et al.. | Ascorbate Attenuates Atrial Pacing-Induced Peroxynitrite Formation And Electrical Remodeling And Decreases The Incidence Of Postoperative Atrial Fibrillation | Circulation Research | 2001 | 403 | 16.79 |
| 65 | Liddle Ad et al.. | Adverse Outcomes After Total And Unicompartmental Knee Replacement In 101 330 Matched Patients: A Study Of Data From The National Joint Registry For England And Wales | Lancet | 2014 | 402 | 36.55 |
| 66 | Gandhi Gy et al.. | Intensive Intraoperative Insulin Therapy Versus Conventional Glucose Management During Cardiac Surgery - A Randomized Trial | Annals Of Internal Medicine | 2007 | 400 | 22.22 |
| 67 | Wesselink Em et al.. | Intraoperative Hypotension And The Risk Of Postoperative Adverse Outcomes: A Systematic Review | British Journal Of Anaesthesia | 2018 | 399 | 57.00 |
| 68 | Coselli Js et al.. | Outcomes Of 3309 Thoracoabdominal Aortic Aneurysm Repairs | Journal Of Thoracic And Cardiovascular Surgery | 2016 | 398 | 44.22 |
| 69 | Taylor Dw et al.. | Low-Dose And High-Dose Acetylsalicylic Acid For Patients Undergoing Carotid Endarterectomy: A Randomised Controlled Trial | Lancet | 1999 | 393 | 15.12 |
| 70 | Lewis Sc et al.. | General Anaesthesia Versus Local Anaesthesia For Carotid Surgery (Gala): A Multicentre, Randomised Controlled Trial | Lancet | 2008 | 392 | 23.06 |
| 71 | Johansson Bb et al.. | Brain Plasticity And Stroke Rehabilitation - The Willis Lecture | Stroke | 2000 | 390 | 15.60 |
| 72 | Cooper Wa et al.. | Impact Of Renal Dysfunction On Outcomes Of Coronary Artery Bypass Surgery - Results From The Society Of Thoracic Surgeons National Adult Cardiac Database | Circulation | 2006 | 387 | 20.37 |
| 73 | Hogue Cw et al.. | Risk Factors For Early Or Delayed Stroke After Cardiac Surgery | Circulation | 1999 | 384 | 14.77 |
| 74 | Myles Ps et al.. | Tranexamic Acid In Patients Undergoing Coronary-Artery Surgery | New England Journal Of Medicine | 2017 | 383 | 47.88 |
| 75 | Noblett Se et al.. | Randomized Clinical Trial Assessing The Effect Of Doppler-Optimized Fluid Management On Outcome After Elective Colorectal Resection | British Journal Of Surgery | 2006 | 378 | 19.89 |
| 76 | Perkins Gd et al.. | Cardiac Arrest And Cardiopulmonary Resuscitation Outcome Reports: Update Of The Utstein Resuscitation Registry Templates For Out-Of-Hospital Cardiac Arrest A Statement For Healthcare Professionals From A Task Force Of The International Liaison Committee On Resuscitation | Resuscitation | 2015 | 375 | 37.50 |
| 77 | Chaturvedi S et al.. | Carotid Endarterectomy - An Evidence-Based Review - Report Of The Therapeutics And Technology Assessment Subcommittee Of The American Academy Of Neurology | Neurology | 2005 | 375 | 18.75 |
| 78 | Marchant Mh et al.. | The Impact Of Glycemic Control And Diabetes Mellitus On Perioperative Outcomes After Total Joint Arthroplasty | Journal Of Bone And Joint Surgery-American Volume | 2009 | 371 | 23.19 |
| 79 | Katz Es et al.. | Protruding Aortic Atheromas Predict Stroke In Elderly Patients Undergoing Cardiopulmonary Bypass - Experience With Intraoperative Transesophageal Echocardiography | Journal Of The American College Of Cardiology | 1992 | 370 | 11.21 |
| 80 | Loef Bg et al.. | Immediate Postoperative Renal Function Deterioration In Cardiac Surgical Patients Predicts In-Hospital Mortality And Long-Term Survival | Journal Of The American Society Of Nephrology | 2005 | 368 | 18.40 |
| 81 | Wennberg De et al.. | Variation In Carotid Endarterectomy Mortality In The Medicare Population - Trial Hospitals, Volume, And Patient Characteristics | Jama-Journal Of The American Medical Association | 1998 | 365 | 13.52 |
| 82 | Karkouti K et al.. | Risk Associated With Preoperative Anemia In Cardiac Surgery - A Multicenter Cohort Study | Circulation | 2008 | 361 | 21.24 |
| 83 | Monk Tg et al.. | Association Between Intraoperative Hypotension And Hypertension And 30-Day Postoperative Mortality In Noncardiac Surgery | Anesthesiology | 2015 | 360 | 36.00 |
| 84 | Biller J et al.. | Guidelines For Carotid Endarterectomy - A Statement For Healthcare Professionals From A Special Writing Group Of The Stroke Council, American Heart Association | Circulation | 1998 | 355 | 13.15 |
| 85 | Bellomo R et al.. | Prospective Controlled Trial Of Effect Of Medical Emergency Team On Postoperative Morbidity And Mortality Rates | Critical Care Medicine | 2004 | 354 | 16.86 |
| 86 | Jacobi J et al.. | Guidelines For The Use Of An Insulin Infusion For The Management Of Hyperglycemia In Critically Ill Patients | Critical Care Medicine | 2012 | 348 | 26.77 |
| 87 | Elbardissi Aw et al.. | Trends In Isolated Coronary Artery Bypass Grafting: An Analysis Of The Society Of Thoracic Surgeons Adult Cardiac Surgery Database | Journal Of Thoracic And Cardiovascular Surgery | 2012 | 348 | 26.77 |
| 88 | Guzman R et al.. | Clinical Outcome After 450 Revascularization Procedures For Moyamoya Disease | Journal Of Neurosurgery | 2009 | 347 | 21.69 |
| 89 | Rossini Pm et al.. | Transcranial Magnetic Stimulation - Diagnostic, Therapeutic, And Research Potential | Neurology | 2007 | 347 | 19.28 |
| 90 | Selim M et al.. | Current Concepts - Perioperative Stroke | New England Journal Of Medicine | 2007 | 341 | 18.94 |
| 91 | Loftus Im et al.. | Increased Matrix Metalloproteinase-9 Activity In Unstable Carotid Plaques A Potential Role In Acute Plaque Disruption | Stroke | 2000 | 336 | 13.44 |
| 92 | Rudolph Jl et al.. | Derivation And Validation Of A Preoperative Prediction Rule For Delirium After Cardiac Surgery | Circulation | 2009 | 335 | 20.94 |
| 93 | Fowler Aj et al.. | Meta-Analysis Of The Association Between Preoperative Anaemia And Mortality After Surgery | British Journal Of Surgery | 2015 | 333 | 33.30 |
| 94 | Healey Js et al.. | Left Atrial Appendage Occlusion Study (Laaos): Results Of A Randomized Controlled Pilot Study Of Left Atrial Appendage Occlusion During Coronary Bypass Surgery In Patients At Risk For Stroke | American Heart Journal | 2005 | 333 | 16.65 |
| 95 | Kondziolka D et al.. | Neurotransplantation For Patients With Subcortical Motor Stroke: A Phase 2 Randomized Trial | Journal Of Neurosurgery | 2005 | 333 | 16.65 |
| 96 | Nathoe Hm et al.. | A Comparison Of On-Pump And Off-Pump Coronary Bypass Surgery In Low-Risk Patients | New England Journal Of Medicine | 2003 | 332 | 15.09 |
| 97 | Braunberger E et al.. | Very Long-Term Results (More Than 20 Years) Of Valve Repair With Carpentier'S Techniques In Nonrheumatic Mitral Valve Insufficiency | Circulation | 2001 | 323 | 13.46 |
| 98 | Scheeren Twl et al.. | Monitoring Tissue Oxygenation By Near Infrared Spectroscopy (Nirs): Background And Current Applications | Journal Of Clinical Monitoring And Computing | 2012 | 322 | 24.77 |
| 99 | Henry DA et al | Anti-fibrinolytic use for minimising perioperative allogeneic blood transfusion | Cochrane Database Syst Rev | 2007 | 322 | 17.88 |
| 100 | Gandhi Gy et al. | Intraoperative hyperglycemia and perioperative outcomes in cardiac surgery patients | Mayo Clin Proc | 2005 | 320 | 16.84 |
